# B cells and monocytes from patients with active multiple sclerosis exhibit increased surface expression of both HERV-H Env and HERV-W Env, accompanied by increased seroreactivity

**DOI:** 10.1186/1742-4690-6-104

**Published:** 2009-11-16

**Authors:** Tomasz Brudek, Tove Christensen, Lars Aagaard, Thor Petersen, Hans J Hansen, Anné Møller-Larsen

**Affiliations:** 1 Department of Medical Microbiology and Immunology, University of Aarhus, DK-8000 Aarhus C, Denmark; 2 Department of Molecular Biology, University of Aarhus, DK-8000 Aarhus C, Denmark; 3 Department of Neurology, University of Aarhus, DK-8000 Aarhus C, Denmark

## Abstract

**Background:**

The etiology of the neurogenerative disease multiple sclerosis (MS) is unknown. The leading hypotheses suggest that MS is the result of exposure of genetically susceptible individuals to certain environmental factor(s). Herpesviruses and human endogenous retroviruses (HERVs) represent potentially important factors in MS development. Herpesviruses can activate HERVs, and HERVs are activated in MS patients.

**Results:**

Using flow cytometry, we have analyzed HERV-H Env and HERV-W Env epitope expression on the surface of PBMCs from MS patients with active and stable disease, and from control individuals. We have also analyzed serum antibody levels to the expressed HERV-H and HERV-W Env epitopes. We found a significantly higher expression of HERV-H and HERV-W Env epitopes on B cells and monocytes from patients with active MS compared with patients with stable MS or control individuals. Furthermore, patients with active disease had relatively higher numbers of B cells in the PBMC population, and higher antibody reactivities towards HERV-H Env and HERV-W Env epitopes. The higher antibody reactivities in sera from patients with active MS correlate with the higher levels of HERV-H Env and HERV-W Env expression on B cells and monocytes. We did not find such correlations for stable MS patients or for controls.

**Conclusion:**

These findings indicate that both HERV-H Env and HERV-W Env are expressed in higher quantities on the surface of B cells and monocytes in patients with active MS, and that the expression of these proteins may be associated with exacerbation of the disease.

## Background

The cause of the inflammatory, neurodegenerative disease multiple sclerosis (MS) remains unknown. Etiological and epidemiological studies suggest that an infectious agent or agents operating on a background of genetic susceptibility are probably involved in the pathogenesis [1]. Among the environmental factors human endogenous retroviruses (HERV) and the ubiquitously present herpesviruses are gaining growing attention, substantiated by an increasing number of reports suggesting their association with MS [2,3]. Recently, we have demonstrated increased cellular immune responses towards different herpesvirus and HERV antigens when they are concomitantly present in lymphocyte stimulation assays [4]. The cellular immune responses were synergistic in character and tended to be higher in MS patients in comparison with healthy controls.

This *in vitro *observation is pertinent only if herpesvirus and HERV antigens are concurrently present *in vivo *in MS patients. Herpesviruses are highly prevalent worldwide and they all cause latent infections that may subsequently become reactivated. HERVs are distributed in many copies throughout the human genome, and are inherited in a Mendelian fashion. Several herpesviruses are capable of HERV activation as previously demonstrated for HERV-K [5,6] and HERV-W [7,8]. We have recently shown that the presence of inactivated herpesviruses can activate expression of HERVs in particle form in PBMCs from MS patients *in vitro*, most probably resulting in the concurrent presence of these two types of virus [9].

It has also been established that HERVs are present in activated form *in vivo *in MS patients. This is based on the demonstration of activated HERV-H [10,11] and MSRV/HERV-W [12,13] - virions - in blood from MS patients, and increased levels of HERV-H, HERV-K, and HERV-W RNA in MS brains [14]. HERV-W Env and Gag proteins have also been found in brain tissue from MS patients [15,16]. Our previous studies of humoral responses have demonstrated elevated levels of antibodies towards HERV-H Gag and Env epitopes in MS sera and cerebrospinal fluid (CSF) [17,18], while others have reported anti-MSRV/HERV-W antibodies in MS sera using a phage-display library of random pentadecapeptides as capture peptides [19]. These authors reported specific reactivity to four mimotopes in MS CSF. Two of these shared similarity with the HERV-W Env sequence [19]. However, we have subsequently found that all four mimotopes have higher similarities to HERV-H Env sequences [2]. Anti-HERV antibody reactivities will presumably be directed towards epitopes on virions as well as on lymphocyte surfaces.

In this manuscript, we present the first evidence that both HERV-H and HERV-W Envs are present at higher levels on the surface of PBMCs from patients with active or stable MS in comparison with PBMCs from healthy and neurological controls. Using flow cytometry, we have analyzed the levels of specific Env epitopes on the surface of different leukocyte populations. As a follow up to our previously published studies we have analyzed serum antibody reactivities towards these particular HERV-H and HERV-W Env epitopes, and correlated these reactivities with Env expression levels.

## Results

### Western Blot and flow cytometric analyses of HERV-H Env and HERV-W Env expression on the surface of cells and particles obtained from MS cell cultures

The polyclonal anti-HERV-H/-W Env TM (transmembrane region) and SU (surface unit region) rabbit antibodies were used in Western Blot analyses of purified retroviral particles from MS1946 cell culture, to detect whether these Env epitopes are present on virion surfaces.

**Figure 1:**
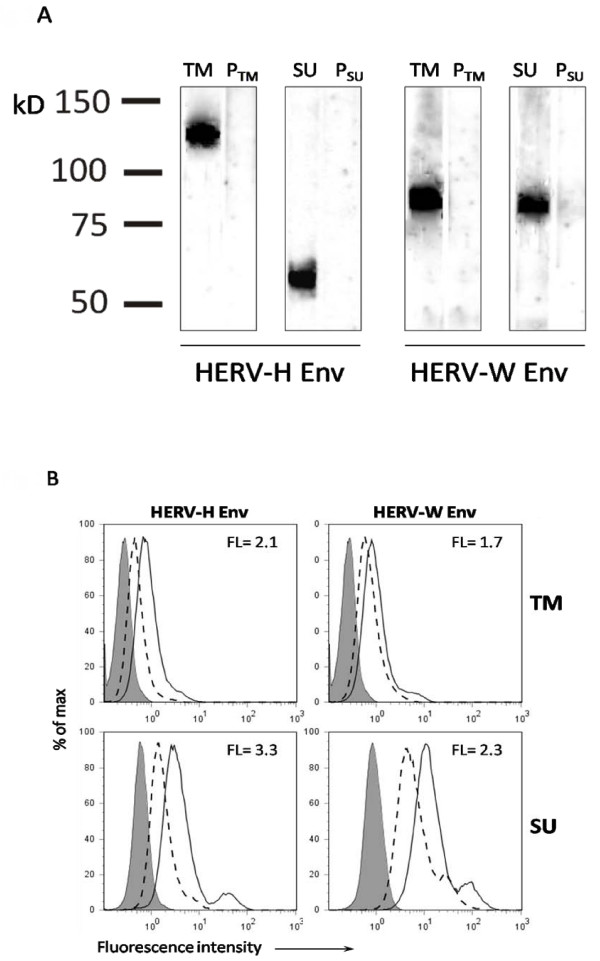
**Western blot analyses of OptiPrep purified HERV particles from MS 1946 long-term, lymphoblastoid cell cultures**. Anti-HERV-H/-W Env TM and SU antibodies were raised in New Zealand white rabbits against 17-mer peptides localised at specific, but equivalent positions in the Env ORFs of HERV-H env62/H19 (Env H1TM: aa489-505; Env H3SU: aa 370-386) and of syncytin 1 (Env W1TM: aa415-431, Env W3SU: aa301-317). Size markers are shown to the left. TM, SU -- anti-HERV Env TM/SU serum; P_TM_, P_SU _-- appropriate pre-immune control sera. **A**. Flow cytometric analysis of surface expression of HERV-H Env and HERV-W Env TM and SU epitopes on cells from MS 1946 long-term lymphoblastoid cell cultures. The grey peaks represent the fluorescence of cells incubated with human IgG; the peaks with dashed line represent fluorescence of the cells incubated with pre-immune serum and FITC goat anti-rabbit antibodies; and the peaks with solid line represent fluorescence of the cells incubated with anti-HERV-H/-W Env TM/SU anti-sera and FITC goat anti-rabbit antibodies. Fluorescence indices are calculated as the ratio of the mean fluorescence of the cells incubated with anti-Env Abs to the mean fluorescence of the cells incubated with the appropriate control (pre-immune serum).

The polyclonal anti-HERV-H/-W Env antibodies were raised towards equivalent but specific peptide epitopes: two peptides were localized in the TM regions of HERV-H and HERV-W Envs, respectively, and two peptides were localized in the SU regions. The results are presented in figure 1A. For HERV-H Env TM, a band of approximate molecular mass of 120 kDa was present, whereas for HERV-H Env SU a band of approximate molecular mass of 60 kDa was found. Bands of approximate molecular masses of 80 kDa, corresponding to both HERV-W Env TM and HERV-W Env SU, were present in virions produced by the MS1946 cell culture. The 60, 80 and 120 kDa bands were absent on blots incubated with the appropriate pre-immune sera. The bands at 60 and 80 kDa are likely to correspond to monomeric glycosylated Envs, while the band at 120 kDa may represent envelope protein aggregates or protein dimers as described for other retroviral Envs [20-23].

The differences in band sizes may be a result of HERV-H/-W Env heterogenecity. At least three different ORFs for HERV-H Env, and one (Syncytin 1) for HERV-W Env, supplemented by a number of sequences with almost intact coding capacity, are dispersed in the human genome [22-27].

To compare the presence of HERV Env epitopes on virions with expression of the same epitopes on cell surfaces of the virion producing cell culture, we performed flow cytometric analyses. The results presented in figure 1B corroborate the Western Blot analyses as the cell culture expresses both HERV-H Env and HERV-W Env epitopes. However, whereas the equivalent HERV-W Env TM and SU epitopes are detectable on the surface of the cells, they are present at markedly lower levels than the HERV-H Env TM and SU epitopes.

### Flow cytometric analysis of HERV-H Env and HERV-W Env expression on PBMCs

PBMCs isolated from patients with active MS, stable MS, from healthy controls and neurological non-inflammatory disease controls were incubated with the anti-HERV-H/-W Env anti-sera (Additional File: Supplementary fig. 1), and also with anti-CD4, anti-CD8, anti-CD14, or anti-CD19 antibodies, allowing a concurrent determination of HERV Env expression, and determination of different leukocyte phenotypes.

The HERV-H Env and HERV-W Env TM and SU epitopes were also found to be present on CD19+ (B cells) as on CD14+ cells (monocytes), whereas we did not detect either HERV-H Env or HERV-W Env epitopes on CD4+ T cells or on CD8+ T cells (data not shown).

**Figure 2:**
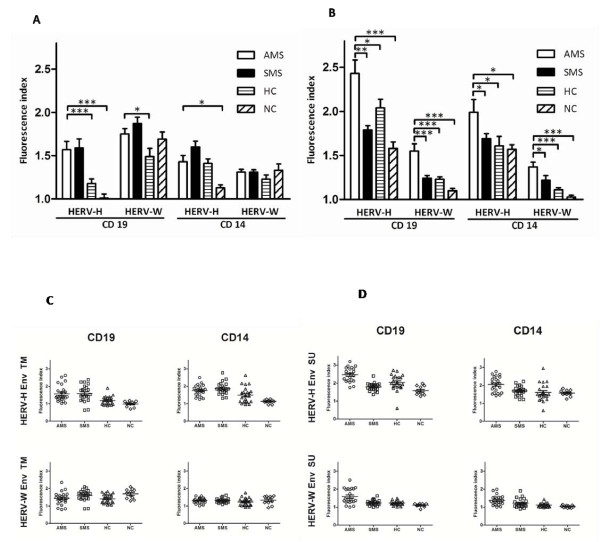
**Flow cytometric analysis of surface expression of HERV-H Env and HERV-W Env on B-cells and monocytes from patients with active MS (AMS), stable MS patients (SMS), healthy individuals (HC), and neurological non-inflammatory controls (NC)**. The results are presented as fluorescence indices calculated as the ratio of the mean fluorescence of the cells incubated with anti-Env Abs to the mean fluorescence of the cells incubated with the appropriate control (pre-immune serum). Mean values (2A) and scatter plots (2C) for cells incubated with anti-Env TM Abs. Mean values (2B) and scatter plots (2D) for cells incubated with anti-Env SU Abs. The standard errors for each group are presented. The significant differences (*P *≤ 0.05) between the groups are shown. * - *P *≤ 0.05; ** - *P *≤ 0.01; ***- *P *≤ 0.001 on column bar graphs 2A and 2B.

The surface expression of HERV-H Env TM epitopes on CD19+ cells was significantly higher in patients with MS, regardless of the disease activity, than in both groups of control individuals (*p *≤ 0.001)(fig. 2A and fig. 2C). The CD19+ cell expression of HERV-W Env TM epitopes was also significantly higher in both group of MS patients but only when compared with healthy controls (*p *= 0.02). CD14+ cell expression of HERV-H Env TM was significantly higher in both MS patient groups compared with neurological non-inflammatory disease controls (*p *≤ 0.05).

Results obtained using anti-Env SU antibodies are presented in figure 2B and 2D. HERV-H Env SU epitope expression was significantly higher on B cells and monocytes in all groups of individuals compared with HERV-W Env SU epitope expression (*p *< 0.01). The surface expression of the HERV-H Env SU epitope on CD19+ cells was significantly higher in patients with active MS than in patients with stable MS (*p *= 0.0001), healthy controls (*p *= 0.04), and neurological controls (*p *= 0.009). The CD19+ cell expression of the HERV-W Env SU epitope was significantly higher in the group of patients with active MS compared with stable MS (*p *= 0.0014), healthy controls (*p *= 0.0008), and neurological controls (*p *= 0.0009). Similarly, on CD14+ cells HERV-H Env SU and HERV-W Env SU epitope expression levels were higher in patients with active MS compared with stable MS patients (for both SU epitopes, respectively *p *= 0.05, *p *= 0.05), healthy controls (for both SU epitopes, respectively *p *= 0.03, *p *= 0.0001), and neurological controls (for both SU epitopes, respectively *p *= 0.05, *p *= 0.0002).

### Characterisation of leukocyte phenotypes

**Figure 3:**
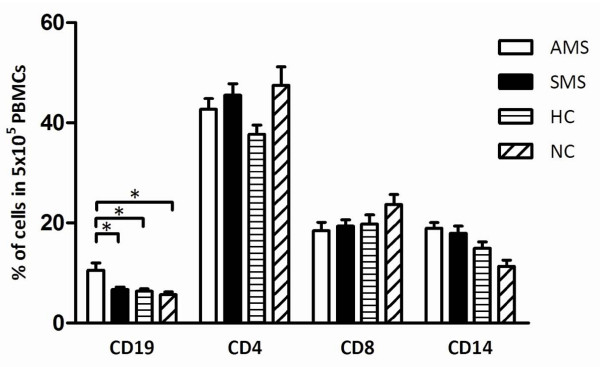
**Flow cytometric analysis of different leukocyte populations in PBMCs isolated from patients with active MS (AMS), stable MS patients (SMS), healthy individuals (HC), and neurological non-inflammatory controls (NC)**. The bars represent the percentage of CD19, CD4, CD8, and CD14 cells in 5 × 10^6 ^PBMCs. The mean values and the standard error for each group are presented, and the significant differences (*P *≤ 0.05) between the groups are shown. * - *P *≤ 0.05; ** - *P *≤ 0.01; ***- *P *≤ 0.001

We characterised the basic leukocyte populations in the PBMC samples from MS patients and healthy controls in parallel with the quantification of HERV-H/-W Env epitope expression on cell surfaces. The results are presented in figure 3.

Patients with active MS had a significantly higher number of B cells compared with patients with stable MS, healthy controls, or neurological non-inflammatory disease controls. We did not find significant differences in the numbers of CD4+ T cells, CD8+ T cells, or monocytes.

### Levels of anti-HERV-H Env and anti-HERV-W Env antibodies

Having demonstrated that the surface expression of HERV-H/-W Env epitopes is higher on B cells and monocytes from patients with active MS, we analyzed the serological reactivity towards these epitopes. Accordingly, the peptides used for capture in this serological screening were identical to the peptides used for rabbit immunisation.

**Figure 4:**
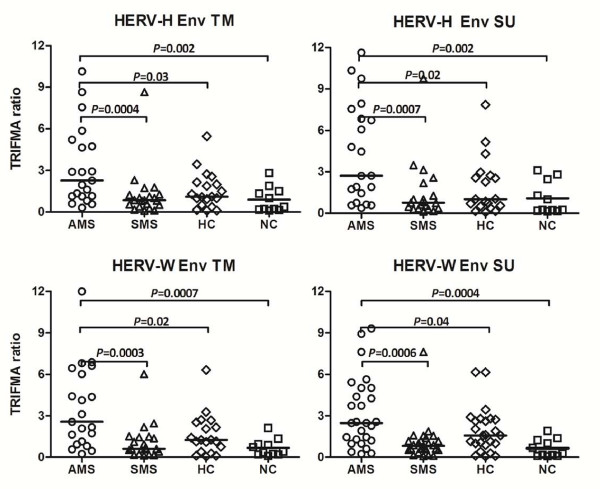
**Seroreactivity to HERV-H Env and HERV-W Env derived peptides in patients with active MS (AMS), stable MS patients (SMS), and healthy individuals (HC) and neurological  non-inflammatory controls (NC).** The horizontal lines indicate median TRIFMA ratios for each group. Significant differences (*P *≤ 0.05) between the groups are shown.

**Figure 5:**
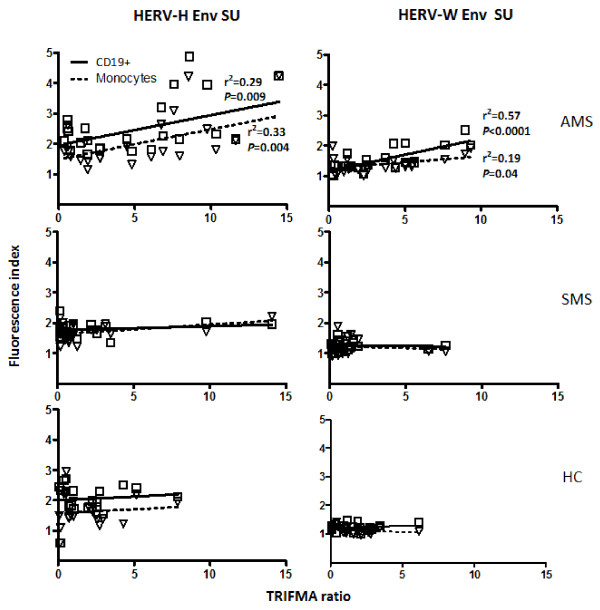
**Multiparameter regression between the levels of anti-HERV-H/-W Env blood serum antibodies measured by TRIFMA vs. the levels of surface expression of HERV-H Env and HERV-W Env epitopes on B-cells and monocytes from patients with active MS (AMS), stable MS patients (SMS), and healthy individuals (HC) measured by flow cytometry (expressed as fluorescence indices calculated as the ratio of the mean fluorescence of the cells incubated with anti-Env Abs to the mean fluorescence of the cells incubated with the appropriate control (pre-immune serum))**. The solid line for CD19+ cells and dashed line for monocytes indicate the regression line. Correlation coefficient (r^2^) and statistical (*P *≤ 0.05) significance are shown.

Figure 4 presents the analysis of the serological reactivity towards the HERV-H/-W Env peptide epitopes in sera from MS patients with active and stable MS as well as from healthy and disease controls. Sera from MS patients with active disease exhibited significantly higher levels of reactivity to all four peptides compared with patients with stable MS and with control individuals. Moreover, the levels of antibodies correlated with the levels of HERV-H/-W Env epitopes expressed on B cells and monocytes from patients with active MS, whereas such a correlation could not be found for stable MS patients, or for either group of control individuals (figure 5).

## Discussion

An increasing number of reports indicate a role for HERVs in MS pathogenesis. The two Gammaretroviruses, HERV-H and HERV-W are activated in MS patients during periods with disease activity [10,13,17,18]. Moreover, previous demonstrations of retroviral RNA in plasma/serum samples from MS patients indicate the presence of HERV virions and/or HERV proteins [10,13].

A previous study has analyzed HERV mRNA levels in brain and lymphocyte populations from 20 MS patients unstratified for disease activity [28]. The present investigation is the first report of concomitant surface expression of both HERV-H Env and HERV-W Env epitopes on PBMCs from MS patients with active or stable MS, and controls. In addition, we have extended the findings by analyses of antibody reactivity towards these epitopes.

Initial investigations using flow cytometry demonstrated the presence of HERV-H Env and HERV-W Env on the surface of cells isolated from long-term MS cell cultures. Virions from the same cultures were analyzed by Western Blotting demonstrating the concomitant presence of both HERV-H and HERV-W Env epitopes in OptiPrep purified virions. This illustrates the known complexity of the HERV particles produced in MS [2]. Moreover, the different sizes of bands in Western Blots suggest individual Env expression patterns for HERV-H and HERV-W. In an earlier study we have demonstrated that Wistar rats, immunised with OptiPrep purified virions produced in long-term MS cell cultures, develop a serological response towards HERV-H Gag and Env derived synthetic peptides [17]. The present results are more direct indications of the presence of HERV Env epitopes on the virions produced in the MS cell cultures.

We also present analyses of the expression of HERV-H Env and HERV-W Env epitopes on the surface of PBMCs from patients with active MS, stable MS, and from control individuals. Both envelope proteins were detected on B cells and monocytes only, with the expression of HERV-H Env epitopes generally higher than the expression of HERV-W Env epitopes. Moreover, there were significantly higher quantities of both Envs on individual cells from patients with active MS compared with patients with stable MS and the control groups.

The apparent differences in the expression of HERV-H/-W Env TM and SU epitopes could be due to distinct features in the molecular Env-membrane interactions [29], or may be inherent in the assay as it could be due to differences in the glycosylation of the envelope proteins. Several of the HERV-H Env precursors are longer than the HERV-W Env (Syncytin 1) precursor and contain more putative N-linked glycosylation sites [20,22,23] which could affect the epitope exposure and thus its interaction with antibodies. An example is a putative glycosylation site at position aa370 in the HERV-H Env SU epitope, as it has previously been shown that glycosylation of HIV Env epitopes can affect their conformation as well as their interaction with antibodies [30].

Antibodies against TM region of HERV Envs have been successfully used in immune assays including flow cytometry and immunofluorescence staining by others, as they are able to detect the TM region of a given Env protein [31-34]. Furthermore, it was shown for anti-HIV Env TM antibodies that they may utilize the cellular membrane to access and bind to gp41 [35], and that the membrane-embedded HIV Envs elicit broader immune responses than soluble forms of Envs [36].

The expression of the Env epitopes exclusively on the surface of B cells and monocytes may be a consequence of the special relationship between HERVs and herpesviruses. It is already established that herpesviruses can activate HERVs [7-9,37-40] and it is likely that reactivated herpesviruses may transactivate HERVs at the transcriptional level. Many indications also exist that herpesviruses may be involved in MS pathogenesis. EBV, HHV-6, and VZV are the strongest candidates for this involvement [41]. It is well established that EBV persists within memory B cells [42], whereas monocytes are a site of latency for HHV-6 [43]. VZV DNA has been demonstrated in MS patient mononuclear cells in connection with disease exacerbation [44]. Noteworthy in this context is that we have previously shown that the concomitant presence of HERV and herpesvirus antigens induces synergistic cell-mediated immune responses [4,45]. It has also been demonstrated by others that patients with active MS have higher specific cellular immunity towards synthetic HERV Env TM peptides than patients with stable MS [46,47].

Concomitantly with the analyses of HERV Env expression, we analyzed the actual composition of the PBMC populations. Significantly higher numbers of B cells were present in the PBMCs from patients with active MS compared with healthy controls. Thus, the higher number of B cells together with the higher expression of HERV-H Env and HERV-W Env epitopes will augment the total amount of HERV Envs in active MS, while HERV Env expression is lower and hence may be down-regulated in patients with stable MS.

It is widely thought that T cells play a central role in MS pathogenesis and they have previously been the main focus of attention, since CD4+ T cells, as well as CD8+ T cells, reactive to myelin antigens, are present in MS patients and seem to be crucial in the development of some types of demyelinating lesions [48-53]. The autoreactive CD4+ T cells in MS may be activated in the periphery and once activated, they can cross the blood-brain barrier (BBB) [54,55]. Furthermore, several features of MS lesions suggest that Th1 mediated immune responses play an important role in the inflammatory process [56]. A prominent expansion of CD8+ memory T cells have also been found in MS CSF and in MS brain tissue [57,58]. However, it is now emerging that other cell types, i.e. B cells, and also other factors are important [59-61]. Unlike activated T cells, B cells do not appear to cross the intact BBB, whereas the occurrence of BBB damage in MS does permit the entry of B cells and antibodies into the CNS [62] presumably augmenting the characteristic intrathecal antibody synthesis found in a majority of MS patients. Furthermore, B-cell activation is associated with a more serious clinical outcome in MS [63], and B cells, plasma cells, and myelin-specific antibodies are present in some MS plaques [64-66]. One of the roles of the T-cells could be regulation of HERV expressing B cells. It is most likely that HERV expression stimulates antibody production, and in conjunction cytotoxic T cells and antibodies may act synergistically in reducing the increased HERV expression, thereby probably diminishing the immune reactivity and thereby also influence the pathogenesis of the actual MS attack.

The role of HERV-expressing monocytes is more uncertain and not extensively investigated, but besides being regulated by CD4+ T cells, these cells are antigen-presenting cells as are the B cells, which may contribute to T-cell reactivity towards the expressed HERV epitopes.

The apparent link between B-cell expansion in PBMCs and increased relapse-activity in MS is particularly interesting in view of the increasing awareness of the importance of B cells in MS pathogenesis (and the evident therapeutic potential in B-cell depletion [67]) although the main focus so far has been the B cells involved in intrathecal IgG synthesis in the CNS [68]. Our current findings are confirmed by reports of an increased number of B cells during MS relapses [61], and significantly increased levels of the B-cell survival promoter APRIL in MS patients [69]. Recently, short-lived plasma blasts were identified as the main effector B cell population involved in active inflammation in MS patients [70].

The elevated levels of HERV-H and HERV-W Env expression on B-cell and monocytes surfaces in samples from patients with active MS found in the present study is also closely reflected in the antibody response to HERV-H and HERV-W Env peptide epitopes. We have demonstrated significantly elevated levels of serum antibodies towards four representative Env peptide epitopes (HERV-H SU and TM, and HERV-W SU and TM) in samples from patients with active MS. Both the TM and SU regions of retroviral Envs are known to elicit serological responses [71], and whereas the amino acid sequences of these peptide epitopes clearly distinguish HERV-H Env from HERV-W Env, they are localised at equivalent positions in HERV-H and HERV-W Envs. This is completely consistent with our previous findings of increased antibody reactivities towards HERV-H/RGH-2 Env and Gag peptides associated with high MS disease activity compared with control groups such as patients with autoimmune diseases, patients with other neurological diseases, or healthy relatives of MS patients [17,18]. In the current study, serological activities actually correlate with the levels of HERV-H/-W Env surface expression on B cells and monocytes. These consistent findings of higher anti-HERV antibody reactivities in the active phases of the disease substantiate a specific immune reactivity to HERVs in MS. Our findings may be paralleled in the chronic progressive, neurological disease HAM/TSP (HTLV-I (Human T-cell Leukaemia Virus) Associated Myelopathy/Tropical Spastic Paraparesis) which is caused by the human exogenous retrovirus HTLV-I. HAM/TSP is characterized by high levels of virus-specific cytotoxic T cells concomitantly with high levels of anti-HTLV-I Env antibodies in patient sera [72].

Apart from MS, HERVs have been implied in a number of other autoimmune disorders. Examples of suggested Gammaretroviral involvement in autoimmunity include HERV-E and HERV-W in psoriasis [31,73], HERV-E in systemic lupus erythematosus (SLE) [74,75], and HRES-1 in SLE [76,77]. The direct and/or indirect roles of HERV-H and HERV-W, the possible interactions between these two HERVs, and between HERVs and herpesviruses in MS, invites further investigations and there are several possible mechanisms by which HERVs could cause MS [2].

Our present results advocate the hypothesis that expressed ORFs from Gammaretroviruses such as HERV-H and HERV-W, as well as the complex interactions of HERV-expressing cells may play a role in MS development.

## Methods

### Blood samples

23 patients with active MS (18 females and 5 males, age 47 ± 11 years), 23 patients with stable MS (14 females and 9 males, age 49 ± 15 years), 22 healthy controls (11 females and 11 males, age 41 ± 13 years), and 11 patients with epilepsy (6 females and 5 males, age 45 ± 13 years) used as controls with an unrelated, neurological disease, were enrolled in the study. Patients with epilepsy comprise a heterogeneous group, often with focal inflammatory reactions as the cause of the seizures, which makes these patients a relevant control group for MS. Moreover, it has become increasingly evident that these inflammatory reactions mediate some of the changes seen in connection with seizures [78-81].

Stringent selection criteria were applied to all MS patients to ensure that only individuals with "typical" and clinically well-characterized relapsing-remitting MS were included in the study. The MS patients were selected at the Neurology Department, Aarhus University Hospital, and gave written informed consent to participate in the study. The Central Denmark Region Committee on Biomedical Research Ethics gave ethical approval for the sampling of blood, culturing of cells from patients, and use in the study. Information about the MS patients is provided in table 1. All MS patients fulfilled the diagnostic criteria of Poser *et al.*, 1983 [82]. Relapsing-remitting (RR) MS is defined in accordance with the Poser criteria as at least two previous relapses in different CNS regions confirmed by neurological examination. Stratification of MS disease activity is also performed according to standard criteria: Active MS is defined as at least one relapse within one year prior to the examination (i.e. high annual relapse rate), while stable MS is defined as the absence of disease activity for at least a year as determined by standard clinical criteria. None of the MS patients or the control individuals had any evidence of an infectious disease within the last 3 months prior to the study.

**Table 1:** Clinical data for MS patients: a -- active MS; RR -- relapsing-remitting; F -- female; M -- male.

**MS patient**	**MS type**	**Age (years)**	**Gender**	**MS patient**	**MS type**	**Age (years)**	**Gender**
	
1	RR a	28	M	24	RR	56	F
2	RR a	70	F	25	RR	59	F
3	RR a	34	F	26	RR	63	F
4	RR a	40	F	27	RR	50	F
5	RR a	53	F	28	RR	65	M
6	RR a	62	M	29	RR	55	M
7	RR a	35	M	30	RR	29	F
8	RR a	56	F	31	RR	63	F
9	RR a	37	F	32	RR	31	M
10	RR a	53	M	33	RR	58	M
11	RR a	30	M	34	RR	23	M
12	RR a	38	F	35	RR	43	M
13	RR a	60	F	36	RR	58	F
14	RR a	54	F	37	RR	66	M
15	RR a	30	F	38	RR	57	F
16	RR a	51	F	39	RR	35	M
17	RR a	53	F	40	RR	39	F
18	RR a	53	F	41	RR	83	F
19	RR a	58	F	42	RR	56	F
20	RR a	51	F	43	RR	40	F
21	RR a	49	F	44	RR	26	F
22	RR a	44	F	45	RR	28	F
23	RR a	60	F	46	RR	43	M

Venous blood was drawn at the respective clinics and processed on the same day in our laboratory. PBMCs were prepared by standard Isopaque-Ficoll centrifugation. The separated cells were cryopreserved in RPMI with addition of 20% human serum (HS) and 10% DMSO, at - 135°C until use.

### Cell cultures

The long-term, lymphoblastoid cell culture MS1946, originating from PBMCs from a patient with active MS, was grown as described previously [83,84]. In brief, the cells were grown at 0.5 × 10^6 ^cells/ml of RPMI-1640, supplemented with 10% inactivated human serum. Cells were split three times a week and supplemented with fresh medium. Twenty four hours before harvest of supernatants, the suspensions were supplemented with additional fresh medium (approx. 30% of the total volume) to obtain optimal growth conditions and thereby optimal virus production. Only batches with sufficiently high retrovirus production as confirmed by PERT (PCR-enhanced reverse transcriptase assay) for reverse transcriptase activity [9,85] were used for virus purification.

### Anti-HERV Env Antibodies

Polyclonal Anti-HERV-H Env and anti-HERV-W Env antibodies were raised in New Zealand White rabbits against 16-mer peptides localised at equivalent positions in the Env ORFs of HERV-H env62/H19 (Env H1TM: aa489-505; Env H3SU: aa 370-386) [22,23] and syncytin 1 (Env W1TM: aa415-431, Env W3SU: aa301-317) [25], respectively. These peptide sequences fulfil the criteria of immunogenicity, and they are localised at equivalent positions in the HERV-H and HERV-W Envs, while having highly dissimilar amino acid sequences. Both the peptides and the anti-sera were prepared by Sigma-Genosys, UK. Preimmune sera were collected before immunisation. Two rabbits were immunised with each peptide, boosted 3 times, and after the final boost, peripheral blood was collected from each rabbit for subsequent measuring of anti-peptide antibodies.

The specificity and cross-reactivity of the anti-HERV-H/-W Env antisera against the peptides were analyzed using TRIFMA assays. The anti-HERV-H Env epitope antisera were at least a 1000 times more reactive towards the HERV-H Env peptides than towards HERV-W Env peptides, and *vice versa *(data not shown).

### Virion purification and Western Blotting

The expression of HERV-H and HERV-W Env epitopes on the virions produced by the long-term MS1946 cell culture was analysed by Western Blotting of purified particles. These virions were purified by ultracentrifugation of 800 ml samples of cell culture supernatants (1.2 × 10^6 ^cells/ml) in self-generating Iodixanol gradients (Nycomed, Norway) as described in detail elsewhere [86], and fractionated. Fractions with high reverse transcriptase activity as measured by PERT were pooled, suspended in TNE (50 mM Tris-HCl pH 7.5, 100 mM NaCl, 1 mM EDTA) with 0.1% HSA (human serum albumin) and stored at -70°C until use.

The pooled virion-containing gradient fractions were loaded onto 4-12% Bis-Tris precast gels, electrophoresed in MOPS buffer (Criterion TM XT system, Biorad, Richmond, CA, USA), and electrophoretically transferred to Hybond nitrocellulose membranes in transferbuffer (25 mM Tris-HCl, 192 mM Glycine, 20% EtOH, 0,1% SDS, pH 8.5). Relative molecular sizes were interpolated from curves constructed on the basis of coloured marker proteins (Biorad Richmond, CA, USA, Precision standard). After incubation with the primary, polyclonal rabbit antibodies (diluted 1/2500) at room temperature overnight, the blots were treated with horseradish peroxidase-labeled secondary goat anti-rabbit antibodies (diluted 1/4000)(DAKO, Denmark) followed by enhanced chemiluminescence reagent (Super Signal West Pico, Pierce Biotechnologies, Rockville, USA). Blots were visualised using a Kodak ID Image Station.

### Flow Cytometric Analysis

Multi-colour flow cytometry was performed to determine both the phenotypes of the cells, and HERV-H Env and/or HERV-W Env cell surface expression. The phenotypes were determined using monoclonal antibodies, anti-CD19-PE (cat.no. 12-0199), anti-CD4-PE-Cy5 (cat.no. 15-0049), anti-CD8-PE-Cy7 (cat.no. 25-0088), and anti-CD14-PE-Cy7 (cat.no. 25-0149) purchased from eBioscience. The anti-HERV-H Env and anti-HERV-W Env antibodies described above were used for HERV-H and HERV-W Env epitope detection, visualised using goat anti-rabbit IgG, F(ab')_2 _conjugated with FITC (PIERCE, cat. no. 31573). Isotype controls included mouse IgG1PE, PE-Cy5, and PE-Cy7 (eBioscience cat. no. 12-4714, 15-4714, 25-4714). Pre-immune sera from the appropriate rabbits were used as controls for the anti-Env antibodies. The monoclonal antibodies were used in concentrations as suggested by the manufacturer. The polyclonal rabbit antibodies were diluted 1/1000 before use. Prior to staining with antibodies all PBMC samples were incubated with human IgG (Statens Serum Institute, Beriglobin, cat. no. 2948) in a concentration of 100 μg/10^6 ^cells/ml to avoid non-specific antibody binding.

Flow cytometry analysis was performed on a Beckman Coulter Cytomics FC500 flow cytometer. The data were analyzed using Flow-Jo v.7 software (Treestar, San Carlos, CA, USA). A total of at least 50,000 events were analyzed for each sample.

The results from the relative quantification of HERV-H Env and HERV-W Env epitope expression are presented as fluorescence (FL) indices, which were obtained by dividing the mean fluorescence of the cells incubated with anti-Env antibodies by the mean fluorescence of cells incubated with the appropriate pre-immune control serum.

### Time-Resolved Immunofluorometric Assay (TRIFMA) for anti- Env peptide antibodies

TRIFMA is a highly sensitive and reliable method for antibody detection, using europium-labelled secondary antibodies. The peptides used for capture were the same as the peptides used for raising the polyclonal anti-HERV-H Env and anti-HERV-W Env rabbit antibodies. The assay was performed essentially as described previously [17,18]. In brief, sera were diluted 1/500 in TBS/Tween and tested in duplicate. The fluorescence was measured using a time-resolved plate fluorometer (LKB, Wallac). Results are presented as TRIFMA ratios, defined as individual measurements in relation to the mean of TRIFMA controls. As TRIFMA inter-assay controls, 8 sera from healthy individuals representing both high and low responders were used [18]. The control sera were included in all TRIFMA assays.

### Statistical analysis

For statistical calculations, Mann-Whitney testing and multiple regression testing were performed using GraphPad Instat ver.3. For TRIFMA, the highest and the lowest responder were excluded from each group before the analysis was performed.

## Competing interests

The authors declare that they have no competing interests.

## Authors' contributions

TB has made substantial contributions to conception, design, and acquisition of data, as well as statistical analysis and interpretation of data. TB carried out the molecular genetic studies, Western Blotting, flow cytometric analyses, and TRIFMA immunoassays. TB wrote a first draft of the paper, with contributions from other authors to the following drafts and the final version. TB has given final approval of the version to be published. TC has made substantial contributions to conception, design, analysis, and interpretation of data. TC has been involved in drafting the manuscript and revising it critically for important intellectual content. TC has given final approval of the version to be published.

LA has made substantial contributions to conception, design, analysis and interpretation of data from molecular genetic studies. LA has given final approval of the version to be published. TP has made substantial contributions to conception of the study. TP has been responsible for enrolment of MS patients and collection of blood samples. TP has given final approval of the version to be published.

HJH has made substantial contributions to conception of the study. HJH has been responsible for enrolment of MS patients and collection of blood samples. HJH has given final approval of the version to be published. AML has conceived and has been the main coordinator of the study. AML has made substantial contributions to conception, design, analysis, and interpretation of data. AML has been involved in drafting the manuscript and revising it critically for important intellectual content. AML has given final approval of the version to be published. All authors read and approved the final manuscript.

## Supplementary Material

Additional file 1 **Supplementary figure 1**. Flow cytometric analysis of surface expression of HERV-H and HERV-W Env epitopes on B-cells and monocytes from patients with active MS (AMS), stable MS patients (SMS), healthy individuals (HC), and neurological non-inflammatory controls (NC). The figure is a presentation of flow cytometric data analyses. From each group, two individuals representing high and low levels of HERV-H/-W Env epitope expression are shown. Blue peak - cells incubated with secondary goat anti-rabbit IgG, F(ab')_2 _FITC; green peak - cells incubated with appropriate pre-immune serum; red peak - cells incubated with anti-HERV-H/W Env TM/SU serum. The numbers in square brackets represent the fluorescence index calculated as the ratio of the mean fluorescence of the cells incubated with anti-Env Abs to the mean fluorescence of the cells incubated with the appropriate control (pre-immune serum). Click here for file
