# Supplementary material for: B cells and monocytes from patients with active multiple sclerosis exhibit increased surface expression of both HERV-H Env and HERV-W Env, accompanied by increased seroreactivity
Source: Retrovirology. 2009 Nov 16;6:104. doi: 10.1186/1742-4690-6-104 (PMC2780989; doi:10.1186/1742-4690-6-104)
Supplement: Additional file 1 — Supplementary figure 1. Flow cytometric analysis of surface expression of HERV-H and HERV-W Env epitopes on B-cells and monocytes from patients with active MS (AMS), stable MS patients (SMS), healthy individuals (HC), and neurological non-inflammatory controls (NC). The figure is a presentation of flow cytometric data analyses. From each group, two individuals representing high and low levels of HERV-H/-W Env epitope expression are shown. Blue peak - cells incubated with secondary goat anti-rabbit IgG, F(ab')2 FITC; green peak - cells incubated with appropriate pre-immune serum; red peak - cells incubated with anti-HERV-H/W Env TM/SU serum. The numbers in square brackets represent the fluorescence index calculated as the ratio of the mean fluorescence of the cells incubated with anti-Env Abs to the mean fluorescence of the cells incubated with the appropriate control (pre-immune serum). [file 1742-4690-6-104-S1.PPT]

## Slide 1
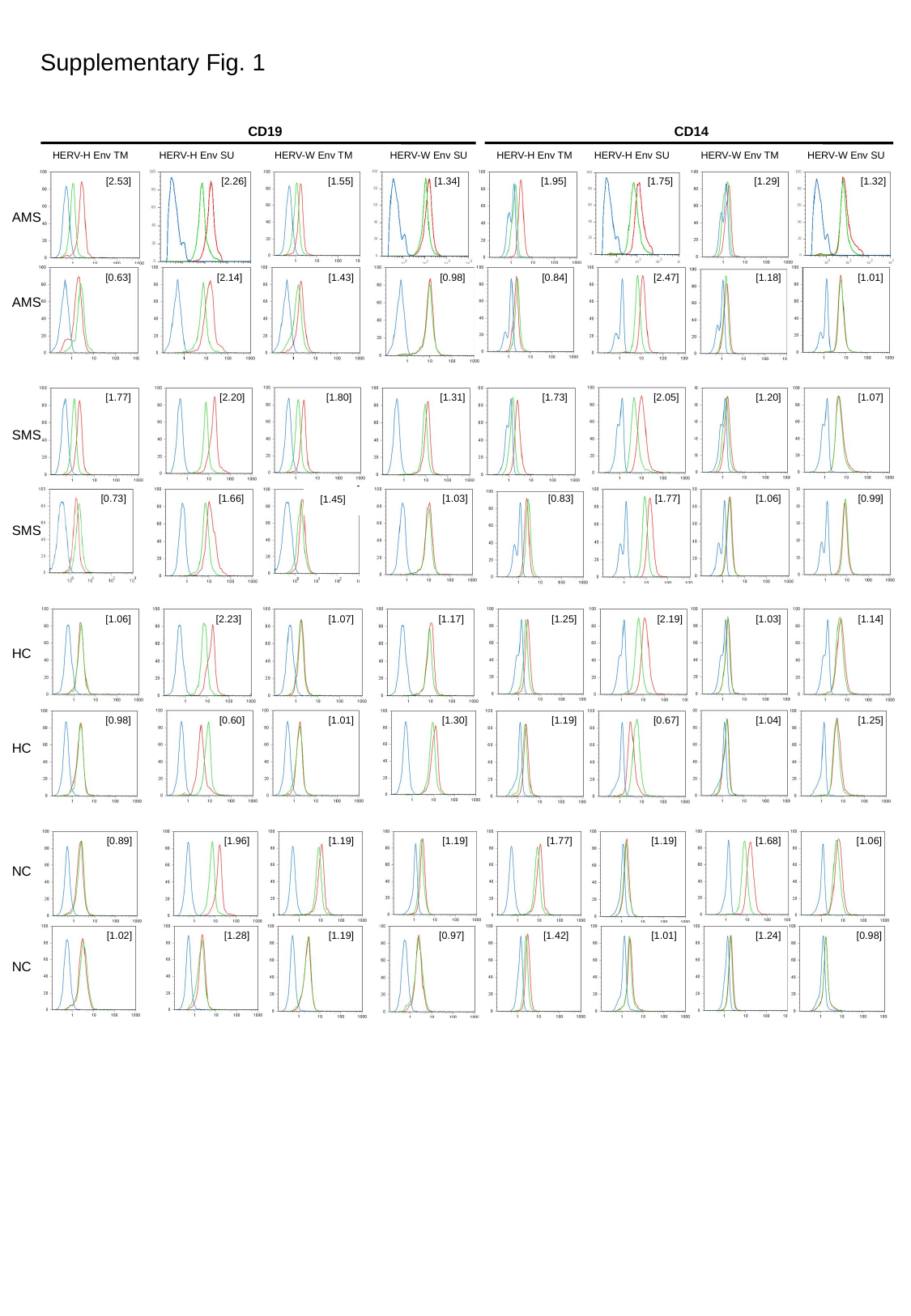

Supplementary Fig. 1
CD19
CD14
HERV-H Env TM
HERV-H Env SU
HERV-W Env TM
HERV-W Env SU
HERV-H Env TM
HERV-H Env SU
HERV-W Env TM
HERV-W Env SU
[2.53]
[2.26]
[1.55]
[1.34]
[1.95]
[1.75]
[1.29]
[1.32]
AMS
[0.63]
[2.14]
[1.43]
[0.98]
[0.84]
[2.47]
[1.18]
[1.01]
AMS
[1.77]
[2.20]
[1.80]
[1.31]
[1.73]
[2.05]
[1.20]
[1.07]
SMS
[0.73]
[1.66]
[1.03]
[0.83]
[1.77]
[1.06]
[0.99]
[1.45]
SMS
[1.06]
[2.23]
[1.07]
[1.17]
[1.25]
[2.19]
[1.03]
[1.14]
HC
[0.98]
[0.60]
[1.01]
[1.30]
[1.19]
[0.67]
[1.04]
[1.25]
HC
[0.89]
[1.96]
[1.19]
[1.19]
[1.77]
[1.19]
[1.68]
[1.06]
NC
[1.02]
[1.28]
[1.19]
[0.97]
[1.42]
[1.01]
[1.24]
[0.98]
NC
